# Central venous access device–associated complication costs in paediatric cancer care

**DOI:** 10.1007/s00520-026-10942-1

**Published:** 2026-07-02

**Authors:** Elouise R. Comber, Ruth Royle, Amanda J. Ullman, Victoria Gibson, Mari Takashima, Samantha Keogh, David D. Eisenstat, Michelle Martin, Andrew S. Moore, Joshua Byrnes

**Affiliations:** 1 Children’s Health Queensland Hospital and Health Service, South Brisbane, QLD Australia; 2 The University of Queensland, Brisbane, QLD Australia; 3 Centre for Applied Health Economics, Griffith University, Brisbane, QLD Australia; 4 Centre for Healthcare Transformation, Queensland University of Technology, Brisbane, QLD Australia; 5 Children’s Cancer Centre, The Royal Children’s Hospital Melbourne, Melbourne, VIC Australia; 6 Department of Paediatrics, University of Melbourne, Melbourne, VIC Australia; 7 Stem Cell Medicine, Murdoch Children’s Research Institute, Parkville, VIC Australia; 8 Monash Children’s Hospital, Clayton, VIC Australia

**Keywords:** Costs and cost analysis, Paediatrics, Paediatric oncology, Vascular access devices, Central venous catheters, CVAD-associated complications

## Abstract

**Purpose:**

Central venous access devices (CVADs) are commonly inserted in paediatric patients requiring cancer treatment and supportive care. However, these devices carry a high risk of complications such as central line-associated bloodstream infections (CLABSI), venous thromboembolism (VTE), and occlusion, and are associated with significant management costs. This study aimed to determine the average cost of each CVAD-associated complication type, per complication event, and investigate which elements are the key cost drivers of these estimates.

**Methods:**

This Australian prospective observational cohort study, embedded within a randomised controlled trial, acquired data from hospital purchasing departments, the Medicare Benefits Schedule Book, the National Hospital Cost Data Collection (NHCDC) Public Sector 2022–2023 Report, and local (state-based) enterprise bargaining agreements. Price per resource unit was multiplied by associated resource use and summed across all resources to estimate the mean complication cost per event. An average cost per complication event for each complication type was then determined.

**Results:**

In a sample of 310 paediatric oncology patients, 101 unique patients experienced 275 CVAD-associated complication events. Of these events, estimated per-event direct costs for CVAD-associated complications were as follows: occlusion (A$2712 (95% CI $1198–$4225)), CVAD-associated venous thromboembolism (A$17,510 (95% CI $5840–$29,181)), confirmed CLABSI (A$38,995 (95% CI $22,394–$55,596)), and suspected CLABSI (A$16,030 (95% CI $12,971–$19,090)).

**Conclusion:**

CVAD-associated complications remain a palpable cost in paediatric cancer care, with CLABSI carrying the highest cost burden. Therefore, investing in preventative care is encouraged to minimise the burden of complication costs to the healthcare system, and reduce the physical and emotional burden on patients and their families. With admission costs comprising a high percentage of complication-associated costs, efficient and effective evidence-based care must be delivered to ensure a shorter length of stay, simultaneously improving the patient experience.

**Supplementary Information:**

The online version contains supplementary material available at 10.1007/s00520-026-10942-1.

## Introduction

Paediatric patients requiring cancer treatment and supportive care typically have a central venous access device (CVAD) inserted into the internal jugular, cephalic, or subclavian vein [1]. This device allows a range of therapies, including chemotherapy, parenteral nutrition, fluids, antibiotics, and other medications to be delivered directly into the bloodstream without repeated venipuncture [1]. Blood may also be drawn from these devices for diagnostic sampling [1]. CVADs are often surgically inserted by an interventional radiologist, surgeon, anaesthesiologist, or specialised vascular access nurse, assisted by ultrasound or other imaging methods [2]. Without complications, these devices can remain in place for the duration of treatment, with a median dwell time of 11 months [1]. Long-term CVADs provide a range of benefits for clinical practice as well as reduced physical and emotional trauma for patients, due to reduced venipuncture; however, they still carry the risk of complications [1]. A range of measures and strategies exist to prevent or reduce the occurrence of these complications. This study investigates the cost implications of three main complication types: central-line associated bloodstream infection (CLABSI), venous thromboembolism (VTE), and occlusion.

A CLABSI is a primary bloodstream infection which occurs when bacteria or other pathogens enter the bloodstream via a CVAD and is estimated to occur in 21.2% of CVADs in paediatric oncology settings [3, 4]. In oncology patients, immunosuppression due to disease and treatment contributes to a higher risk of CLABSI [5]. The long-term nature of CVADs, alongside frequent, often intermittent use, also increases infection risk due to increased opportunities for microbial contamination [5]. CLABSIs are commonly treated with antibiotics or other antimicrobials; however, in some circumstances, the line may need to be replaced, delaying life-saving treatment. CLABSI significantly impacts patient outcomes and healthcare costs, contributing to thedevelopment of antibiotic resistance and mortality globally [6]. Thrombosis occurs due to the inflammation and irritation of the vein from the insertion and continued dwell of a CVAD, with treatment options including systemic anticoagulant medications such as heparin or warfarin [7]. Literature estimates venous thromboembolism to occur at a rate of 5.2% in paediatric oncology patients [3]. Occlusion is the blockage of a CVAD, with literature estimating a rate of 6.3% for complete occlusion and 10.2% for all occlusion events [3]. Flushing the line with normal saline is typically sufficient; however, if the line is fully occluded, it may need to be replaced to continue treatment. The risk of both VTE and occlusion is increased in cancer patients due to some systemic anti-cancer therapies, as well as tumour biology, leading to a hypercoagulable state affecting all three elements of Virchow’s triad (blood, vessel wall, and flow) [8]. Other complications such as line fractures, dislodgement, migration, or localised skin infections can also occur but are not included in this analysis [9].

Although CVAD-associated complication costs have been previously estimated, uncertainty exists as to the direct costs in highly vulnerable populations such as paediatric cancer care. A recent review highlighted limited reporting of the methods used for calculating cost estimates of CVAD-associated complications in paediatrics, and substantial heterogeneity in published CVAD-associated complication cost estimates likely attributable to differences in the types of costs included in estimates [10]. Costing data allows the full impact of complications on the health system to be understood. Cost estimates can act as a ‘price-signal’ attracting the attention of all stakeholders in the health system to develop and implement this solution. By calculating the cost of CVAD-associated complications, hospitals can identify the magnitude of this cost and the savings which would ensue from minimising this cost.

## Methods

### Objectives

The following objectives were addressed in this study:

- Determine the direct incident cost burden for each CVAD-associated complication (CLABSI, occlusion, VTE) in paediatric cancer care per event.
- Determine which elements are the key cost-drivers of this burden, by CVAD-associated complication type.

### Design

An Australian prospective observational cohort study was performed to extract and analyse relevant information obtained from the trial database of participants enrolled in a large multi-site clinical trial (ANZCTRN: 12622000499785, registration date: 29 March 2022) [11]. Participant data were included if the participant had concluded their participation in the trial, and therefore complete data were available. This study used a micro-costing approach to determine the cost of CVAD-associated complications in paediatric cancer care, and follows the Consolidated Health Economic Evaluation Reporting Standards (CHEERS) [12].

### Setting, participants and recruitment

All participants included in this study were recruited as part of a larger clinical trial across six Australian hospital sites exploring the efficacy of three catheter locking solutions [11]. Data were obtained from this pooled RCT dataset. Participants were aged 18 years or less with cancer or a malignant haematological condition with a CVAD currently inserted. The International Classification of Childhood Cancers (ICCC) system was used to classify cancer diagnoses [13]. CVADs included peripherally inserted central catheters (PICCs), tunnelled lines, and totally implanted CVADs. Participants were excluded if they had end-of-life measures in place at recruitment, a pre-existing coagulopathic condition not related to their current treatment/diagnosis, or a known allergy to a study intervention. Participants were continuously recruited at all study sites with study participation for 3 months from recruitment or until CVAD removal (whichever occurred first). Data collection for this sub-study began on 5 September 2022 and concluded on 5 September 2025.

### Data collection

Data were collected for the primary study regarding the presence, diagnostic processes, and management of confirmed CLABSI, suspected CLABSI, VTE, and occlusion. All potential CLABSIs were reviewed by an infectious disease specialist blinded to study intervention group allocation. A confirmed CLABSI was determined following identification of an eligible organism in a CVAD in place for more than 2 consecutive days on the day of the first positive blood culture (day of CVAD placement being day 1), unrelated to infection at another site [11, 14]. A suspected CLABSI was defined as a patient with the symptoms of a confirmed CLABSI, including positive blood culture, and treatment commenced by clinicians, without meeting all official CLABSI criteria [14]. VTE was defined as a symptomatic thrombosed vessel with a CVAD inserted, or a fibrin sheath occluding the CVAD lumen [15]. Diagnosis of thrombosis was confirmed by venography or ultrasound. Symptomatic VTE was characterised by pain and/or swelling of the device insertion area or associated catheter occlusion [11]. An occlusion was defined as a complete injection and/or aspiration catheter occlusion of one or more lumens (determined using the Catheter Injection and Aspiration Classification System) and/or a visible split in the catheter related to the occlusion event [11, 16]. The complication event was considered to conclude when no further treatment, scans, or associated hospital admissions were required for the complication.

Clinical data collected in the broader clinical trial [11] used for this economic analysis were stored in the trial database. Data were prospectively collected by trained research nurses from direct observation and patient medical records. Only resources associated with the complication were incorporated regarding complication diagnosis and management. Resources included pharmaceuticals, imaging, blood cultures, hospital staff (for diagnosis and management), consumables, and other resources associated with hospital admission and hospital in the home services (Supplementary Table 1). The standard acute day-rate [17] was used for the cost per hospital admission day. The majority of data was acquired from hospital purchasing departments and was used alongside The Medicare Benefits Schedule [18] and IHACPA National Hospital Cost Data Collection (NHCDC) Public Sector 2022–2023 Report [17]. Where staff costs were required to determine the cost of procedures, local (state-based) enterprise bargaining agreements were used [19, 20]. The minimal amount of data which could not be sourced from these avenues was sourced from the Pharmaceutical Benefits Scheme (two medications), or hospital wholesalers (three consumable items). Supplementary Table 2 displays all resources, costs (actual and inflation adjusted), and their sources.

### Data analysis

Data regarding the occurrence and management of CVAD-related complications were extracted from the trial database and used alongside financial information to determine the average cost per event for CVAD-associated complications as a whole and per complication type. Price per resource unit was multiplied by associated resource use and summed across all resources to estimate the cost per CVAD-associated complication event. The cost of each complication event was estimated, stratified by complication type, and then averaged across each complication type. Average costs were reported alongside 95% confidence intervals for each CVAD-associated complication (occlusion, VTE, confirmed CLABSI, suspected CLABSI). Costs associated with incidental diagnoses were not included in cost estimates.

The expected costs per complication were compared to determine which complication carried the greatest incident cost burden. For the purpose of analysis, complications were treated as individual events (e.g. if one patient had multiple complications over the duration of the study period, these were viewed separately).

Costs were grouped into four main categories: imaging/diagnostic costs, pharmaceuticals, management procedures, and hospital admission. Hospital admission costs included any costs related to bed use, staff wages associated with monitoring and delivering care, and administrative costs. Any testing, scans, procedures, or pharmaceuticals administered during the hospital stay have been allocated to the appropriate category and are not included in the hospital admission cost. Comparison of resource costs across the different CVAD-associated complications was explored to identify complication-specific cost drivers. All costs are presented in 2025 Australian dollars (A$), adjusted for inflation using the Reserve Bank of Australia’s Inflation Calculator [21] from the perspective of the Australian publicly funded healthcare system.

## Results

Data from a total of 310 participants were analysed, revealing 101 patients with 275 CVAD-associated complication events. All participants were paediatric patients with an oncological diagnosis, recruited as part of the broader clinical trial. There was a relatively even split between gender and age group (except infants). The largest proportion of participants were patients of the Queensland Children’s Hospital (*n* = 191, 62%) and were diagnosed with leukaemia or a myeloproliferative or myelodysplastic disease (*n* = 169, 55%). A large proportion of participants had been recently diagnosed (≤ 30 days) when recruited for the trial (*n* = 122, 39%) (Table 1). Of the 101 unique participants who experienced a CVAD-associated complication, a large number had either one (*n* = 33, 33%) or two (*n* = 27, 27%) complications.

**Table 1:** Participant demographics (*N* = 310)

Characteristic	*n* (%)
Gender	
Male	167 (54%)
Female	143 (46%)
Age (at study commencement)	
< 1 year	3 (1%)
1 to 2 years	51 (16%)
3 to 5 years	97 (31%)
6 to 11 years	73 (24%)
12 to 18 years	86 (28%)
Study site	
Queensland Children’s Hospital	191 (62%)
Royal Children’s Hospital Melbourne	66 (21%)
Monash Children’s Hospital	20 (6%)
Gold Coast University Hospital	13 (4%)
Sydney Children’s Hospital	11 (4%)
Sunshine Coast University Hospital	9 (3%)
Diagnosis	
Leukaemia, myeloproliferative and myelodysplastic diseases	169 (55%)
Lymphomas and reticuloendothelial neoplasms	36 (12%)
CNS and miscellaneous intracranial and intraspinal neoplasms	27 (9%)
Malignant bone tumours	22 (7%)
Soft tissue and other extraosseous sarcomas	14 (5%)
Renal tumours	12 (4%)
Neuroblastoma and other peripheral nervous cell tumours	10 (3%)
Germ cell tumours, trophoblastic tumours and neoplasms of gonads	7 (2%)
Retinoblastoma	5 (2%)
Hepatic tumours	4 (1%)
Other and unspecified malignant neoplasms	2 (1%)
Diagnosis not stated	2 (1%)
Length of time since diagnosis (at study commencement)	
≤ 30 days	122 (39%)
1–6 months	94 (30%)
6 to 12 months	44 (14%)
1 to 2 years	36 (12%)
> 2 years	14 (5%)
Number of complications per patient (***n*** = 101)	
1	33 (33%)
2	27 (27%)
3	13 (13%)
4	13 (13%)
≥ 5	15 (15%)

Of the participants who experienced a CVAD-associated complication, 60 (*n* = 130 events) experienced an occlusion, 7 experienced a VTE, 8 experienced a confirmed CLABSI, and 69 (*n* = 130 events) experienced a suspected CLABSI. The estimated average direct mean cost per event for each complication type is displayed in Table 2. Confirmed CLABSIs carried the highest cost burden at A$38,995 (95% CI $22,394–$55,596), with occlusion carrying the lowest cost burden at A$2712 (95% CI $1198–$4225). Suspected CLABSI carried the highest total cost at A$2,083,948.

**Table 2:** Cost estimates per CVAD-associated complication type

Complication type	Number of patients experiencing a complication (% of *N* = 310)	Number of complication events	Average cost per event (95% CI), A$	Total cost, A$
Occlusion	60 (19.4%)	130	2712 (1198–4225)	352,496
VTE	7 (2.3%)	7	17,510 (5840–29,181)	122,571
Confirmed CLABSI	8 (2.6%)	8	38,995 (22,394–55,596)	311,957
Suspected CLABSI	69 (22.3%)	130	16,030 (12,971–19,090)	2,083,948

Table 3 shows confirmed CLABSIs represent the highest mean number of hospital days (10.1 days) and blood cultures (4 cultures). Venous thromboembolism (VTE) carried the highest number of imaging episodes (1.6 imaging episodes). Line replacements were highest for VTE (*n* = 4, 57.1%) and confirmed CLABSI (*n* = 4, 50%).

**Table 3:** Key metrics for each CVAD-associated complication

	Occlusion (*n* = 130)	VTE (*n* = 7)	Confirmed CLABSI (*n* = 8)	Suspected CLABSI (*n* = 130)
Hospital days (mean number per event)	**0.6**	**4**	**10.1**	**3.8**
Paediatrics hospital admission	0.6	4	9	3.6
Hospital in the home	0	0	1.1	0.2
PICU	0*	0	0	0*
Blood cultures (mean number per event)	0	0	4	2.7
Imaging episodes (mean number per event)	0.2	1.6	0	0
Line removals/replacements (n) (%)	5 (3.8%)	4 (57.1%)	4 (50%)	7 (5.4%)

*One patient was admitted to PICU for 2 days

**Fig. 1:**
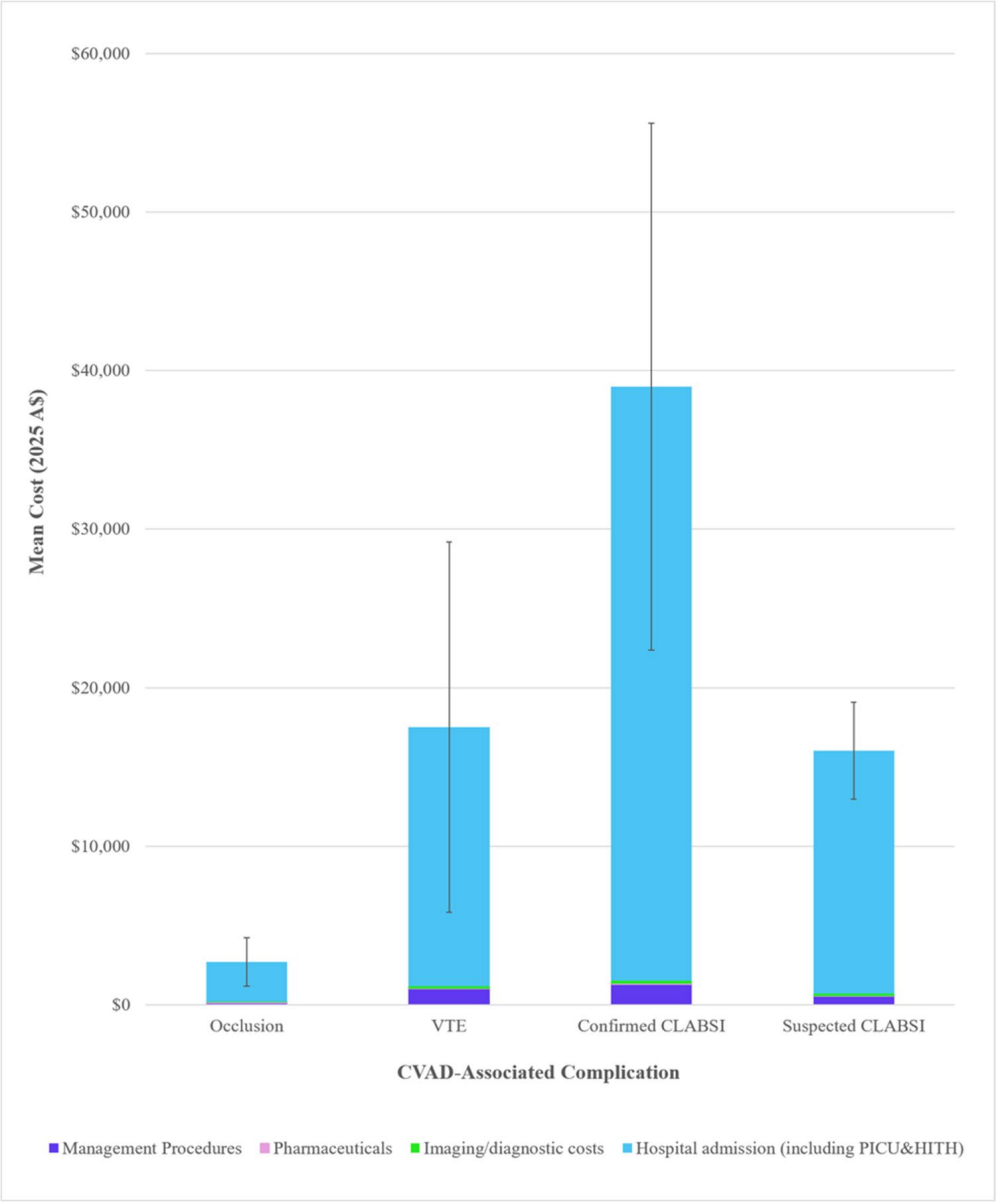
Cost types included in complication cost estimates per event (2025 AUD). Error bars indicate 95% confidence intervals. CLABSI, central line-associated bloodstream infection; PICU, paediatric intensive care unit; HITH, hospital in the home

Figure 1 displays each complication type, and the types of costs which comprise each complication. Hospital admission costs were the main cost (> 92% of total cost per event) for all complication types. Management procedures for VTE and confirmed CLABSI carried higher costs per event than occlusion and suspected CLABSI. Imaging/diagnostics had the highest mean cost per event in the confirmed CLABSI group. Pharmaceutical costs were low across all complication types.

## Discussion

This study highlighted the two most frequently occurring CVAD-associated complications—occlusion and suspected CLABSI. Confirmed CLABSI was identified to carry the highest per event cost burden, with suspected CLABSI and VTE also carrying high costs. Understanding these costs emphasises the need for hospitals to change practice to reduce the occurrence of these complications. CVAD-associated complications lead to an increase in length of stay which carries a significant cost. If complications are reduced, this cost can be avoided or minimised. Other care options such as hospital in the home may also reduce costs whilst still allowing for management and surveillance of less severe complications. However, it is important to note that an extended length of stay may be necessary as treatment or surgery cannot continue until the infection or complication is cleared. The cost burden of hospital admission highlights not only the interruption to treatment but also the likelihood of causing hospital-wide flow-on effects such as waiting lists and bed block.

This study included a significant number of occlusion events (*n* = 130 events in 60 patients (19%)), much higher than previously reported rates of 10.2% [3]. This slightly higher rate may be representative of higher rates of haematological malignancies (leukaemia) in the population, influencing hypercoagulability and coagulopathy [1]. Younger patients or patients with previous VTEs also have an increased risk of occlusive events, as well as multi-lumen CVADs, tunnelled CVADs, and ports [3, 22]. However, CVAD-associated VTE (2.3%) and confirmed CLABSI (2.6%) occurred at significantly lower rates than those reported in Nunn et al.’s 2024 meta-analysis [3] (5.2% and 21.2%, respectively).

Whilst occurring at a relatively low rate, confirmed CLABSI held the highest mean cost per event (A$38,995 (95% CI $22,394–$55,596)), primarily driven by an increased length of stay and need for continuous monitoring to prevent escalation. Previous literature has reported CLABSI costs in paediatrics at A$17,932 [9], and higher in paediatric oncology populations at A$68,202 and A$141,768 (converted to 2025 AUD for comparison) [23, 24]. Whilst per event confirmed CLABSI carried the highest cost, it is important to note that the high volume of suspected CLABSI cases led to a significant overall cost of almost A$2.1 million, whilst the total cost for the eight confirmed CLABSI events was much lower at ~ A$312,000. Patients with CLABSI often require 1–2 weeks of antibiotics to target these infections, requiring close monitoring for treatment resistance [25]. Flow-on complications such as sepsis must also be closely monitored, allowing for intervention with organ dysfunction, fluid resuscitation, and PICU escalation [26]. Additional sequelae such as abscesses, endocarditis, or osteomyelitis require close monitoring and management, also often delaying discharge [26]. Infected lines often need to be removed and replaced which adds further additional costs, including staff, anaesthesia, and consumables (incorporated in the current cost analysis) [26]. Although lower than for confirmed cases, the cost of suspected CLABSI remained substantial (A$16,030 [95% CI $12,971–$19,090]), largely due to shorter treatment periods and reduced per event hospital admission costs. Nevertheless, the proportion of expenditures attributable to pharmaceuticals was comparable. The clinical challenge is to reduce costs associated with suspected CLABSI by shortening time to exclusion. Diagnostic costs were slightly higher in the suspected group, due to the increased number of blood cultures to continuously monitor for CLABSI development. Therefore, diagnostic costs may be reduced by revising the monitoring protocol for suspected CLABSI and reducing the frequency of blood cultures if deemed safe and appropriate. Kumar et al.’s [27] prospective observational study highlights a non-invasive diagnostic approach with paired blood cultures and differential time to positivity to distinguish line infection from other bacteraemia sources, allowing for faster exclusion of CLABSI. Approaches such as these are important avenues to explore further to reduce the cost burden associated with investigating CLABSI.

While occlusion was one of the most frequently occurring complications, it held the lowest per event cost (A$2712 (95% CI $1198–$4225)). Occlusion can usually be managed in routine clinical care with thrombolytic therapy such as alteplase, and rarely prolongs hospital stay. Imaging and diagnostic costs also remain low as detection typically occurs via a clinical assessment of failure to aspirate or inject into the catheter. Whilst the lowest cost of all complications, occlusion costs in this study are much higher than previously published occlusion cost estimates in paediatrics potentially due to more thorough care protocols and higher Australian staff wages [9]. VTE also carries a relatively low cost (A$17,510 (95% CI $5840–$29,181)) compared to confirmed CLABSIs (A$38,995 ($22,394–$55,596)). Management procedures for VTE take a larger proportion than for other complications (5.8%). These procedures typically include presentations to the emergency department due to pain and the need for catheter replacement. Future work should aim to focus on processes and interventions which decrease complications, to minimise LOS and the financial burden on the healthcare system. This analysis considers direct healthcare costs only and does not capture the potential downstream impact of complications on treatment delivery. In paediatric oncology, catheter-related complications may lead to interruptions in therapy, which have been associated with reduced treatment intensity and, in some contexts, poorer clinical outcomes. As such, the overall burden of these complications may be underestimated.

### Strengths and limitations

This study generates cost estimates for the major CVAD-associated complications in a paediatric cancer care population. Methods are detailed clearly and all data sources and inclusions are detailed in the main body of the text and supplemental material. The authors ensured this transparency due to findings in a previous systematic review which highlighted a lack of methods reporting in previous paediatric CVAD costing studies [10]. As this study was undertaken alongside an active RCT, no missing data were apparent as data could be validated from the patient’s medical records.

Although this study collected data from hospital sites around Australia, there is still a skew towards data collected at the Queensland Children’s Hospital (62%) since the RCT initially commenced at this site. The dominance of this site may limit the generalisability of these findings both within Australia and internationally. If CVAD-associated complication prevention procedures were better (or worse) at this hospital, this may skew incidence rates and therefore associated costs and limit generalisability. As this study was undertaken alongside an active RCT, there is the potential the intervention decreased the cost of complications in this study. Assumptions made when analysing data have been detailed in Supplementary Table 2 which may have influenced cost outcomes. No data was clearly omitted, and it was assumed that clinicians entered all medications and management procedures directly associated with the complication (i.e. other procedures unrelated to the complication were not included and no interventions associated with the complication were missed). It is also important to note that delays to chemotherapy (due to CVAD-associated complications) carry significant costs. However, this could not be directly costed in this study apart from additional length of stay. It is important to consider the far-reaching impact of CVAD complications and the bounty of indirect costs which are also associated with these complications including other forms of value such as quality of life and emotional distress. Whilst we acknowledge the importance and impact of these indirect costs such as travel and time off work for parents, these costs were outside the scope of the study which was focused on the costing perspective of the healthcare system. Therefore, costs reported in this analysis likely underestimate the true value of preventing CVAD-associated complications. Whilst this study did not explore differences in complications costs for different device types, this is an interesting topic which may be explored in future studies.

## Conclusion

This study highlights the high costs associated with CVAD complications in paediatric oncology, particularly regarding confirmed CLABSIs. Significant costs were also associated with suspected CLABSIs and VTE. Therefore, redirecting costs to preventative care is encouraged to reduce their occurrence and minimise the burden on the healthcare system, and the physical and emotional burden on patients and their families. Preventative measures such as chlorhexidine dressings and alternative catheter locking solutions have proven particularly beneficial and show great promise for reducing costs associated with CVAD complications [28–30]. With admission costs comprising most complication-associated costs, the most efficient and effective evidence-based care must be delivered to patients to ensure a shorter length of stay, simultaneously improving the patient experience and minimising the indirect effects of hospital admission.

## Supplementary Information

Below is the link to the electronic supplementary material.

ESM 1 Supplementary Material 1 (DOCX 34.4 KB)

## Data Availability

All data is presented in the supplementary material of this manuscript. If additional clarification of data inputs is required, the corresponding author can be contacted – e.comber@uq.edu.au.
