# Supplementary material for: Central venous access device–associated complication costs in paediatric cancer care
Source: Support Care Cancer. 2026 Jul 2;34(7):718. doi: 10.1007/s00520-026-10942-1 (PMC13328129; doi:10.1007/s00520-026-10942-1)
Supplement: Supplementary file 1 — Supplementary Material 1 (DOCX 34.4 KB) [file 520_2026_10942_MOESM1_ESM.docx]

**Supportive Care in Cancer**

**Central Venous Access Device-Associated Complication Costs in Paediatric Cancer Care**

Elouise R Comber, Ruth Royle, Amanda J Ullman, Victoria Gibson, Mari Takashima, Samantha Keogh, David D Eisenstat, Michelle Martin, Andrew S Moore, Joshua Byrnes

**Corresponding Author:**

Elouise Comber

e.comber@uq.edu.au

Children's Health Queensland Hospital and Health Service, South Brisbane, Queensland, Australia.

The University of Queensland, Queensland, Australia.

**Supplementary Tables**

**Supplementary Table 1 – Trial database data extraction variables for the cost of CVAD-associated complications**

| Complication/Topic of Interest | Variable/Question | Variable Response Option |
| --- | --- | --- |
| Occlusion | Management of recent occlusive events | - None - Unknown - Cough/ deep breathing /reposition patient - Turbulent flush - NAD change - CXR/ Line Study - Urokinase - Other |
|  | Occlusion management: Medical Imaging | - Line Study - Xray - CT - Ultrasound - Other |
| Venous Thromboembolism (VTE) | VTE - Investigations undertaken | - Ultrasound - Line study - ECG - Blood culture - Other - None |
|  | Ultrasound/ Diagnostic Imaging - number of investigations | - Free text box (number) |
|  | Anticoagulants to treat thrombosis  Were antimicrobials commenced on suspicion of BSI? | - Already on anticoagulant therapy - IV Heparin - Subcutaneous Low molecular weight heparin - Oral anticoagulation - Other - None |
| CLABSI | Medical imaging for investigation of suspected local infection | - Ultrasound - CT - Xray - MRI - Line Study - Other |
| Economic Outcome of Complications | Did the complication result in: | - None - Chemotherapy delayed (if greater than 1 day) - Delay to discharge (if greater than 1 day) - Emergency Department presentation - Hospital readmission - Hospital in the home admission - Interhospital transfer - OPD presentation/review - Surgical review - Surgical procedure (also select delay to discharge or readmission as required) - Catheter removal - New CVAD - Peripheral venous access device insertion or venipuncture - Dressing change+/_ specialised dressing - Vascular access review - Other specialised nurse review - Oral analgesia - IV analgesia - Port re-needle - Other |
| Delay to Discharge | Delay to discharge - number of days | - Free text box (number, Min: 1) |
|  | Was the patient admitted to PICU after discharge was delayed? | - Yes - No |
|  | Number of days in PICU | - Free text box (number) |
| Readmission | Hospital readmission - was the patient admitted to PICU? | - Yes - No |
|  | Hospital readmission - Number of days in PICU | - Free text box (number, Min: 1) |
|  | Hospital readmission - number of days (general ward - excluding days spent in PICU) | - Free text box (number, Min: 1) |

CLABSI – Central Line Associated Bloodstream Infection; CT – Computed Tomography; CVAD – Central Venous Access Device; CXR – Chest X-ray; ECG – Electrocardiogram; IV – Intravenous; MRI – Magnetic Resonance Imaging; NAD - Nicotinamide Adenine Dinucleotide; OPD – Outpatient Department; PICU – Paediatric Intensive Care Unit; VTE – Venous Thromboembolism.

**Supplementary Table 2 – Cost breakdown and data sources for cost estimates**

| Item | Cost (AUD) | Inflation adjusted cost (2025) | Calculation method | Source |
| --- | --- | --- | --- | --- |
| Imaging/Diagnostics |  |  |  |  |
| Ultrasound - Vascular | $84.75 | $104.11 | - | Item number: 55224  Medicare [1] |
| X-ray | $246.55 | $302.87 | - | Item number: 15500  Medicare [1] |
| Blood culture | $30.75 | $37.77 | - | Item number: 69354  Medicare [1] |
| Management Procedures | | | | |
| CVC removal (<10 years) | $269.75 | $331.37 | - | Item number: 34540  Medicare [1] |
| CVC removal (≥10 years) | $207.50 | $254.90 | - | Item number: 34530  Medicare [1] |
| CVC insertion (< 10 years) | $359.75 | $441.93 | - | Item number: 34534  Medicare [1] |
| CVC insertion (≥10 years) | $560.45 | $688.47 | - | Item number: 34527  Medicare [1] |
| Cough/ deep breathing/ repositioning/ press on port | $0.80 | - | 1 minute - RN or CN | Correspondence with hospital staff or procurement department & State-based enterprise bargaining agreements (Queensland) [2] |
| Dressing Change | $40.86 | - | 30 minutes – RN or CN  Consumables:  5 x Welland removal wipes - $3.15  Sorbaview shield (contour) - $4.88  Dressing pack - $0.51  Cavilon - $1.97  Needleless access device - $1.02  2 x Swabstick (CHG and alcohol) - $0.94  3 x Posiflush - $2.76  Needle (drawing up) - $0.09  Non-sterile gloves - $0.08  Disposable apron - $0.16  Sterile gloves - $1.25 | Correspondence with hospital staff or procurement department & State-based enterprise bargaining agreements (Queensland) [2] |
| NAD Change | $14.05 | - | 15 minutes – CN or RN  Consumables:  Posiflush - $0.92  Needle (drawing up) - $0.09  Needleless access device - $1.02 | Correspondence with hospital staff or procurement department & State-based enterprise bargaining agreements (Queensland) [2] |
| Reneedled Port | $48.94 | - | 30 minutes – CN or RN  Consumables:  5 x Welland removal wipe - $3.15  Sorbaview shield (contour) - $4.88  Dressing pack - $0.51  Cavilon - $0.93  Port needle - $10.14  2 x Swabstick (CHG and alcohol) - $0.94  3 x Posiflush - $2.76  Needle (drawing up) - $0.09  Non-sterile gloves - $0.08  Disposable apron - $0.16  Sterile gloves $1.25 | Correspondence with hospital staff or procurement department & State-based enterprise bargaining agreements (Queensland) [2] |
| Turbulent Flush | $9.03 | - | 10 minutes – CN or RN  Consumables:  Posiflush - $0.92  Needle (drawing up) - $0.09 | Correspondence with hospital staff or procurement department & State-based enterprise bargaining agreements (Queensland) [2] |
| OPD Review (by vascular specialist) | $16.22 | - | 15 minutes – Vascular access CN or NP | Correspondence with hospital staff or procurement department |
| ED Presentation | $980 | $1,039.73 | - | IHACPA [3] |
| Hospital admission, per day (pediatrics) | $3851.86 | $4,086.63 | - | IHACPA [3] |
| PICU admission, per day | $6454.97 | - | - | Correspondence with hospital staff or procurement department |
| Hospital in the home, per day | $600 | - | - | Hospital public-facing data [4] |
| Patient transport service - regional | $656 | - | - | Hospital public-facing data [5] |
| PIVC Insertion | $63.21 | - | 20 minutes – CN or RN  Consumables total: $27.22 (no cost breakdown available) | Correspondence with hospital staff or procurement department & State-based enterprise bargaining agreements (Queensland) [2] |
| Nursing Review | $48.09 | - | 1 hour (average) - CN or RN | Correspondence with hospital staff or procurement department & State-based enterprise bargaining agreements (Queensland) [2] |
| Surgical Review | $116.31 | - | 1 hour – vascular access surgeon | Correspondence with hospital staff or procurement department & State-based enterprise bargaining agreements (Queensland) [6] |
| Vascular Access Review | $32.43 | - | 30 minutes – vascular access CN or NP | Correspondence with hospital staff or procurement department & State-based enterprise bargaining agreements (Queensland) [6] |
| Clinical Staff Wages |  |  |  |  |
| Clinical Nurse (76 hour fortnightly hourly wage) | $54.4487 (band 1) | - | - | State-based enterprise bargaining agreements (Queensland) [2] |
| Registered Nurse (76 hour fortnightly hourly wage) | $41.7355 (band 1) | - | - | State-based enterprise bargaining agreements (Queensland) [2] |
| Nurse Practitioner (76 hour fortnightly hourly wage) | $75.2776 (band 1) | - | - | State-based enterprise bargaining agreements (Queensland) [2] |
| Vascular Access Surgeon (76 hour fortnightly hourly wage) | $116.31315 (midpoint L18-L27) | - | - | State-based enterprise bargaining agreements (Queensland) [6] |
| Infectious Disease Specialist (76 hour fortnightly hourly wage) | $127.8925 (midpoint L25-L27) | - | - | State-based enterprise bargaining agreements (Queensland) [6] |
| Pharmaceuticals (per IV vial or oral dose) | | | | |
| Alteplase (1mg/mL) | $97.65 | - | - | Correspondence with hospital staff or procurement department |
| Ambisome IV 50mg (amphotericin B) | $122 | - | - | Correspondence with hospital staff or procurement department |
| Amikacin IV 500mg/2mL (Medsurge) | $10 | - | - | Correspondence with hospital staff or procurement department |
| Amoxicillin IV 1g (Ibiamox) | $3.30 | - | - | Correspondence with hospital staff or procurement department |
| Amphotericin 10mg oral (Fungilin lozeners) | $4 per bottle | - | - | Correspondence with hospital staff or procurement department |
| Azithromycin 500mg IV (AFT) | $3.60 | - | - | Correspondence with hospital staff or procurement department |
| Azithromycin 500mg oral tablet (Apo-azithromycin) | $3 per box | - | - | Correspondence with hospital staff or procurement department |
| Cefazolin 1g IV (AFT Pharmaceuticals) | $0.80 | - | - | Correspondence with hospital staff or procurement department |
| Cefepime 1g IV (AFT Pharmaceuticals) | $3 | - | - | Correspondence with hospital staff or procurement department |
| Ceftriaxone 1g IV (AFT Pharmaceuticals) | $0.60 | - | - | Correspondence with hospital staff or procurement department |
| Ciprofloxacin 200mg/100mL IV infusion (Aspen) | $7 | - | - | Correspondence with hospital staff or procurement department |
| Clindamycin IV600mg/4mL (Viatris) | $7 | - | - | Correspondence with hospital staff or procurement department |
| Clonidine (MZ) IV 150microg/mL | $6.20 | - | - | Correspondence with hospital staff or procurement department |
| Oxycodone 5mg tablet (Mayne Pharma): 1 x box of 20 tabs = $2.5 | $2.50 | - | - | Correspondence with hospital staff or procurement department |
| Endone (Oxycodone) 5mg | $2.20 | - | - | PBS (subsidised hospital rate) [7] |
| Ertapenem IV 1g (Invanz) | $35 | - | - | Correspondence with hospital staff or procurement department |
| Fentanyl 100microg/2L IV (GH) | $0.71 | - | - | Correspondence with hospital staff or procurement department |
| Flucloxicillin (250mg dose) | $2.38 | - | - | PBS (subsidised hospital rate) [8] |
| Fluconazole 100mg/50mL IV (Diflucan) | $11 | - | - | Correspondence with hospital staff or procurement department |
| Gentamicin 80mg/2mL IV (Pfizer) | $1 | - | - | Correspondence with hospital staff or procurement department |
| Ketamine 200mg/2mL IV (Claris/Baxter) | $5.4 | - | - | Correspondence with hospital staff or procurement department |
| Lincomycin 600mg/2mL IV (SXP) | $8 | - | - | Correspondence with hospital staff or procurement department |
| Meropenem 1g IV (Juno) | $3.90 | - | - | Correspondence with hospital staff or procurement department |
| Metronidazole 500mg/100mL IV (Kabi) | $2 | - | - | Correspondence with hospital staff or procurement department |
| Micafungin 50mg IV (Mycainee) | $80 | - | - | Correspondence with hospital staff or procurement department |
| Morphine 30mg/mL IV (DBL) | $1.80 | - | - | Correspondence with hospital staff or procurement department |
| Paracetamol 1g/100mL IV (Kabi) | $1.60 | - | - | Correspondence with hospital staff or procurement department |
| Paracetamol 50mg/mL (Dymdon) | $4 per bottle | - | - | Correspondence with hospital staff or procurement department |
| Piperacillin Tazobactam IV 4g/0.5g (PipTaz-AFT Pharmaceuticals) | $3 | - | - | Correspondence with hospital staff or procurement department |
| Rivaroxaban 2.5mg tablet (Xarelto) | $0.30 per dose | - | - | Correspondence with hospital staff or procurement department |
| Teicoplanin IV 400mg (Targocid) | $16 | - | - | Correspondence with hospital staff or procurement department |
| Valaciclovir 500mg oral (RBX) | $0.40 | - | - | Correspondence with hospital staff or procurement department |
| Vancomycin 500mg IV (Baxter) | $2.60 | - | - | Correspondence with hospital staff or procurement department |
| Voriconazole 200mg IV (Voriconazole AFT) | $16 | - | - | Correspondence with hospital staff or procurement department |

CN – Clinical Nurse; CVC – Central Venous Catheter; ED – Emergency Department; IHACPA - Independent Health and Aged Care Pricing Authority; IV – Intravenous; NAD - Nicotinamide Adenine Dinucleotide; NP – Nurse Practitioner; PICU – Paediatric Intensive Care Unit; PIVC – Peripheral Intravenous Catheter; OPD – Outpatient Department; RN – Registered Nurse.

**Assumptions:**

- If CN or RN was needed for a procedure, the average of the two wage rates was taken
- If >5 blood cultures was stated, 6 was used as a conservative estimate
- If the delay to discharge was stated as ‘greater than 1 day’ but was not mentioned, 2 was used as a conservative estimate
- For IV medications, one vial per patient was assumed
- For oral medications, one dose per day for the duration of their hospital stay was assumed
- For medications with varying strengths, the most commonly used strength was assumed

**References**

1. Australian Government Department of Health and Aged Care (2020) Medicare Benefits Schedule. <https://www.mbsonline.gov.au/internet/mbsonline/publishing.nsf/Content/Downloads-202001>. Accessed 30 November 2025.

2. Queensland Health (2025) Wage rates - Nursing Stream. Wage rates - Nursing Stream. <https://www.health.qld.gov.au/hrpolicies/wage-rates/nursing>. Accessed 10 December 2025.

3. Independent Health and Aged Care Pricing Authority (2023) National Hospital Cost Data Collection (NHCDC) Public Sector 2022–23. <https://www.ihacpa.gov.au/resources/national-hospital-cost-data-collection-nhcdc-public-sector-2022-23>. Accessed 30 November 2025.

4. The Royal Children's Hospital Melbourne (2025) Your Guide to the RCH. <https://www.rch.org.au/info/az_guide/medicare-ineligible-patient/>. Accessed 11 November 2025.

5. The Victorian Government Department of Health (2025) Ambulance Fees. <https://www.health.vic.gov.au/patient-care/ambulance-fees>. Accessed 11 November 2025.

6. Queensland Health (2025) Wage rates – Medical stream. <https://www.health.qld.gov.au/hrpolicies/wage-rates/medical>. Accessed 10 December 2025.

7. Australian Government Department of Health Disability and Ageing (2025) OXYCODONE. <https://www.pbs.gov.au/medicine/item/13233L-13234M>. Accessed 11 November 2025.

8. Australian Government Department of Health Disability and Ageing (2025) FLUCLOXACILLIN. <https://www.pbs.gov.au/medicine/item/9149M-9150N>. Accessed 11 November 2025.
